# The Impact of Neighbours and Neighbourhoods on Major Depressive Disorders in Adults

**DOI:** 10.1007/s11524-025-01055-x

**Published:** 2026-02-10

**Authors:** Anna Tort-Carrera, J. Paul Elhorst, Govert E. Bijwaard

**Affiliations:** 1 Netherlands Interdisciplinary Demographic Institute (NIDI)–Royal Netherlands Academy of Sciences (KNAW), The Hague, The Netherlands; 2 Faculty of Economics and Business, University of Groningen (RUG), Groningen, The Netherlands

**Keywords:** MDD, Social interaction effects, Linear-in-means model, Lifelines

## Abstract

Major Depressive Disorder (MDD) is a significant global health concern, yet the influence of social interactions among individual neighbours, in addition to neighbourhood characteristics, remains underexplored. This study applies linear-in-means models and spatial econometric techniques to examine whether and which social interactions influence MDD among adults. The data used relate to two repeated cross-sections of 19,700 individuals aged 23 to 65 years collected via the MINI neuropsychiatric interview in two waves of the Lifelines Cohort Study in Northern Netherlands, supplemented with neighbourhood-level information from Statistics Netherlands. Three types of social interactions — peer, contextual, and correlated — are assessed, with Bayesian comparison tests identifying contextual and correlated effects as the most influential. Subsequently, a two-step regression model is employed to address the high prevalence of zero outcomes. The results highlight the influence of contextual and correlated effects among individual neighbours, in addition to individual and neighbourhood characteristics. These findings emphasise the need for further research into social interactions on MDD and support for public health interventions that take them into account.

**Supplementary Information:**

The online version contains supplementary material available at 10.1007/s11524-025-01055-x.

## Introduction

Major Depressive Disorder (MDD) is a significant public health concern with increasing global prevalence [1]. It is characterised by a persistently low mood for most of the day, along with a loss of interest in nearly all activities [2]. MDD has been linked to economic losses in human capital, higher healthcare costs, various mental and physical health conditions, poor quality of life, and even premature mortality [1, 3].

Research on the determinants of MDD shows that its susceptibility and prevalence are shaped by cognitive and biological factors [4]. MDD also exhibits a social gradient where females, ethnic/racial minority groups and individuals with lower education or Socio-Economic Status (SES) face not only a higher risk of developing MDD but also experience more severe consequences [5, 6]. A key factor in this social gradient are neighbourhood effects, which can either amplify or mitigate the risk of MDD, depending on the social context to which individuals are exposed [7, 8]. For instance, lower SES groups often face greater exposure to adverse neighbourhood conditions, including limited medical resources and greater exposure to stressors, all of which contribute to higher MDD prevalence [9]. Although there is growing evidence on the role of the neighbourhood, the mechanisms underlying these associations continue to raise questions, particularly how the same neighbourhood effects affect different population groups in different ways [10].

This lack of clarity has led to a turning point in the neighbourhood effects literature, with increasing calls to move beyond broad associations and towards identifying the mechanisms that underpin these effects [11, 12]. Within this body of research, four main categories of mechanisms have been proposed: social interaction, geographical, environmental, and institutional [13]. Although these mechanisms are widely recognised, empirical studies often fall short of explicitly testing their pathways, particularly in relation to social interaction effects among individual neighbours [14]. Given their theorised role in shaping mental health outcomes, this paper focuses on social interaction effects as another key mechanism influencing MDD [15]. Specifically, this study investigates how social interaction effects, alongside neighbourhood effects, contribute to MDD among adults in the Northern Netherlands. Using data from the Lifelines Cohort Study [16], we apply linear-in-means models and group interaction matrices of individuals living in the same neighbourhood to identify these effects. Our findings offer empirical support for the relevance of social interaction effects in shaping MDD outcomes. These findings could guide targeted policy interventions to address MDD disparities within Dutch communities.

## Social Interactions

The neighbourhood, defined by its residents, their interactions and shared resources and norms, is a key contributor to MDD. Research shows that a favourable social environment alleviates the risk of developing MDD and mediates the role of the neighbourhood-built environment [17]. It is therefore important to consider not only the neighbourhood context itself but also the characteristics of nearby neighbours and how these influence outcomes.

In line with Tobler’s First Law of Geography — “*Everything is related to everything else, but near things are more related than distant things*” [18] — social interactions are typically categorised into peer, contextual and correlated effects [19], which indicate whether and how an individual’s MDD is influenced by the mental health status of nearby neighbours.

Peer effects arise when individuals mutually influence each other’s behaviours [20]. In relation to MDD, they can be understood through the lens of social contagion theory, which posits that both positive and negative emotions can spread through social networks in ways similar to infectious diseases [21]. This process may occur unconsciously through mechanisms such as automatic mimicry [22], or more consciously, via exposure to shared social norms and emotional climates [20]. While existing research has shown that depressive symptoms can spread within social networks [23], it has scarcely been investigated whether this extends to MDD.

Contextual effects refer to the influence of other individuals’ exogenous characteristics, i.e., the SES of nearby neighbours on somebody’s behaviour. In the context of MDD, such effects are particularly relevant because higher average levels of education or income among neighbours are often associated with increased social cohesion, a known protective factor for MDD [24]. Stronger social cohesion can foster a sense of trust, mutual support, and collective efficacy, all of which can buffer against psychological distress and reduce the risk of developing MDD [17].

Lastly, correlated effects acknowledge that an individual behaviour is influenced by unobserved shared characteristics within a neighbourhood. A key example relevant to MDD is neighbourhood stigmatisation, where negative societal perceptions of a neighbourhood become internalised by its residents, potentially leading to chronic stress, diminished self-esteem, and feelings of social exclusion [25], all of which are important to MDD. While much of the existing literature on correlated effects has been theoretical or qualitative in nature, recent studies highlight the need for more empirical research [26, 27].

Using linear-in-means models and spatial econometric techniques, this paper extends previous studies by examining which and the extent to which social interaction effects influence MDD.

## Methodology

### Data Sources

We use data from the Lifelines Cohort study, a large-scale cohort study and biobank focused on risk factors for multifactorial diseases in the population of Northern Netherlands [16]. The study is conducted in accordance with the principles of the Declaration of Helsinki and was approved by the Medical Ethics Committee of the University Medical Centre Groningen (the Netherlands) under number 2007/152.

For this study, we included participants aged 23–65 who participated in both the 2007–2013 and 2014–2017 data collection waves and provided complete information on their MDD assessment (see Figure A1). To enable identification and reliable estimation of social interaction effects, we further restricted the sample to individuals living in neighbourhoods with at least ten participants. In addition, we linked each participant to neighbourhood-level data from Statistics Netherlands (CBS), the lowest scale level at which it collects this data. This resulted in two sets of 19,700 observations across 671 neighbourhoods in 49 municipalities. Table A1 provides an overview of these municipalities. We analyse these observations both as repeated and separate cross-sections.

### MDD and Covariates

**Fig. 1:**
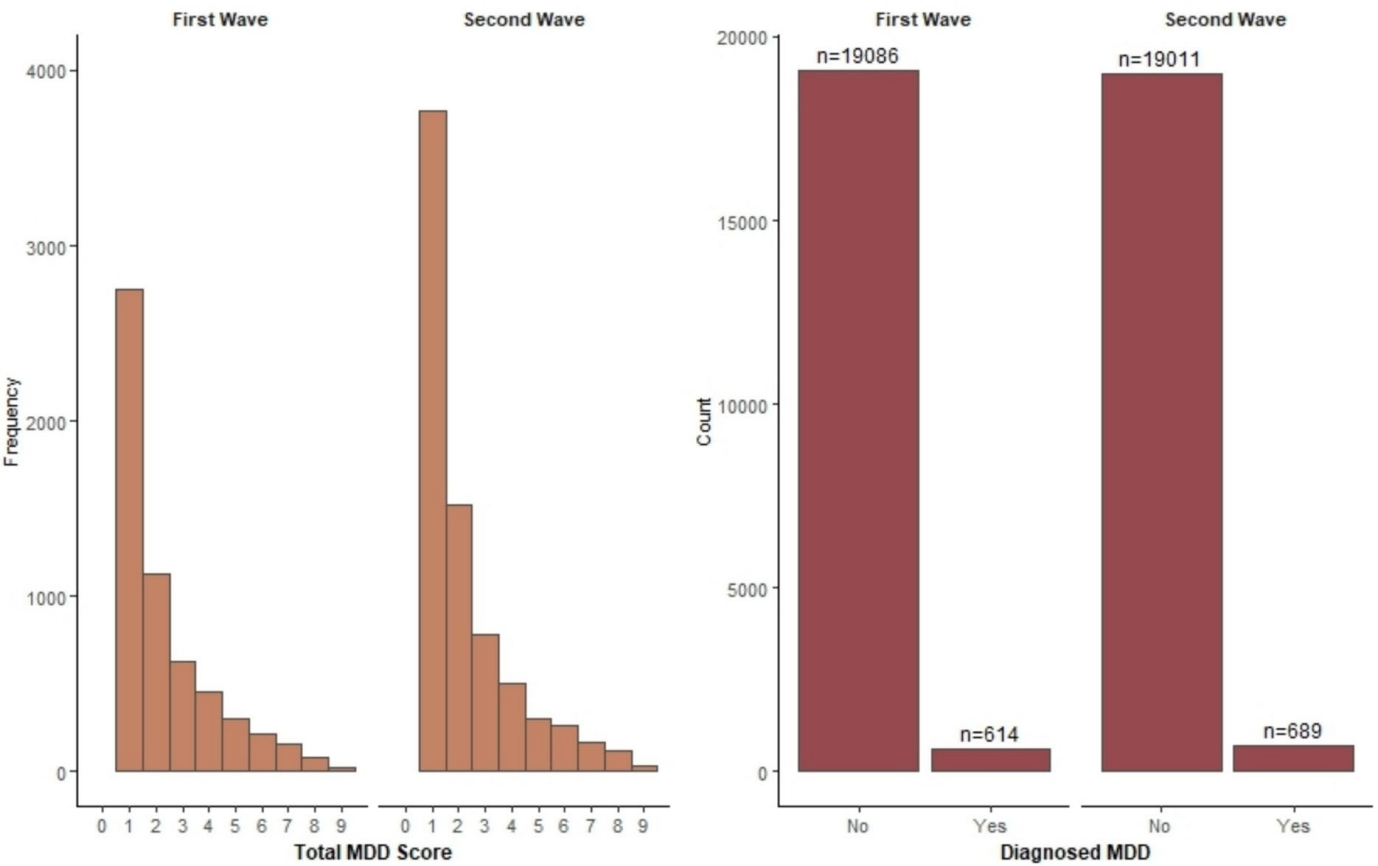
Distribution of total MDD score (left panel) and prevalence of diagnosed MDD (right panel) in both waves of the same 19,700 participants

The presence of a MDD episode (within the past two weeks) is measured using the Mini International Neuropsychiatric Interview (MINI). This is a brief, validated diagnostic tool for Axis I psychiatric disorders, including depression, anxiety, and substance use, based on DSM-IV criteria [2]. In the first wave, referred to below as the baseline assessment, trained medical staff conducted the interviews; in the second wave, the MINI was administered digitally on-site. Participants are asked nine questions, which they can answer with Yes (= 1) or No (= 0). A diagnosis of MDD requires at least one core symptom, either persistent low mood or loss of interest, and four out of seven secondary symptoms, namely changes in appetite, sleep disturbances, fatigue, low concentration, excessive guilt, and suicidal ideation (see Table A2). The left panel of Fig. 1 shows that most participants report low total MDD scores, and the right panel that as a result only 614 participants in the first wave and 689 participants in the second wave were diagnosed with MDD, of whom 384 were among the 19,700 participants in both waves.

MDD is usually modelled as a function of individual and neighbourhood characteristics. We selected those identified in previous studies and available in the Lifelines dataset.

Individual-level characteristics included are age, sex, educational attainment, migration background, children and partner status, depression history, social circle quality, average paid working hours per week, and smoking behaviour. Sex is categorised as Female or Male. Educational attainment is divided into three levels: Low (up to junior general secondary education), Middle (secondary vocational or senior general secondary education), and High (higher vocational or university education). Migration background is classified by country of birth as either within or outside the Netherlands. Partner status is determined based on cohabitation: living with a partner or not in a relationship/not cohabiting. Children status is coded similarly: living with children, without children/not cohabiting. The quality of a participant’s social circle over the past year is categorised as Poor (high stress due to relationship complications), Fair (some stress), or Good (positive relationships). Lastly, smoking behaviour is categorised as Yes (smoked in the current or past month) or No (otherwise).

Neighbourhood-level characteristics included are the average house value as a primary proxy for neighbourhood SES, the percentage of Western and non-Western population as a proxy for the ethno-cultural composition of the neighbourhood, unlike [8] which used it as a proxy for neighbourhood SES, the average number of general practitioners within 3 km, the percentage of single-person households, and the level of urbanity, subdivided into highly urban (≥ 2500 addresses/km^2^), urban (1000–2500 addresses/km^2^), and rural (≤ 500–1000 addresses/km^2^).

Given the relatively low percentage of missing information (< 20%), we performed multiple imputation. Furthermore, continuous characteristics (age, paid working hours per week and all neighbourhood characteristics except urbanity) are scaled by dividing them by 100 to facilitate interpretation in the subsequent regression analyses.

The construction of the peer and contextual effects is based on the individual characteristics and described mathematically in the next section. Although social interaction effects are often interpreted as neighbourhood effects [28], we treat them as conceptually distinct: social interaction effects reflect the average characteristics of other individuals in the same neighbourhood [19], while neighbourhood effects refer to area-level characteristics. To keep them apart, we refer to them below as effects and characteristics, respectively. To be able to estimate the impact of these effects, a network matrix is needed describing the interactions between the participants. Because this network is not observed, and a complete description of the interaction between participants cannot be modelled because they are not all sampled, several researchers use a group interaction matrix as an alternative. In this matrix, participants within the same geographical area (in this study, neighbourhoods) are assumed to interact with each other but not with participants outside this area [29].

## Analysis

To determine which and the extent to which social interaction effects affect MDD, we depart from a standard linear regression model extended with peer, contextual and correlated effects [19, 30]:

1 $${Y}_{jt}={\rho {W}_{j}{Y}_{jt}+X}_{jt}\beta +{W}_{j}{X}_{jt}\theta +{{Z}_{jt}\gamma + \mu }_{j}+{\xi }_{t}+{\varepsilon }_{jt},$$

where ${Y}_{jt}$ is an (${n}_{j}\times 1$) vector of MDD of all individuals ${n}_{j}$ in neighbourhood *j* ($j=1,\dots ,J$) and wave *t* (*t* = 1,2)*.* ${X}_{jt}$ is an (${n}_{j}\times K$) matrix of characteristics observed at the individual level and ${Z}_{jt}$ an (${n}_{j}\times M$) matrix of characteristics observed at the neighbourhood level. Their impact is measured by the parameter vectors $\beta$ and $\gamma$, respectively. The peer effect of MDD with parameter $\rho$ is denoted by ${W}_{j}{Y}_{jt}$ and the contextual effects of the individual characteristics with the parameter vector $\theta$ by ${W}_{j}{X}_{it}$, where ${W}_{j}$ represents the group interaction matrix of each neighbourhood. Since the number of individuals in a neighbourhood is ${n}_{j}$, the off-diagonal elements of *W*_*j*_ are $1/({n}_{j}-1)$, while the diagonal elements are set to zero based on the argument that an individual cannot affect itself and to avoid the so-called reflection problem [19, 31]. Contextual effects of the neighbourhood characteristics are not included, as these are constant across individuals within the same neighbourhood and would result in perfect multicollinearity (${W}_{j}{Z}_{jt}={Z}_{jt}$). The random effects ${\mu }_{j}$ control for correlated effects among individuals living in the same neighbourhood, as well as neighbourhood-invariant variables that may affect MDD, but which are not observed. Additionally, time-invariant factors are controlled for by including wave random effects ${\xi }_{t}$. Lastly, ${\varepsilon }_{jt}$ represents an (${n}_{j}\times 1$) vector of error terms.

Although the social interaction parameters of this model when using a group interaction matrix are identified from an econometric–theoretical perspective [32, 33], their estimation is often problematic due to over-parameterisation [31, 33]. To assess which social interaction effects are empirically relevant, we therefore first apply a Bayesian comparison approach [34]. In this comparison, we use uniform non-informative priors for the social interaction parameters of the peer and correlated effects defined on their parameter space. This allows the posterior model probabilities to be driven by the data rather than by subjective prior information. The Bayesian comparison approach evaluates competing model specifications by their log marginal likelihoods after integrating out all model parameters, with higher values indicating higher posterior model probabilities [34]. The most plausible model specification is the one with the highest posterior probability.

To account for the right-skewed distribution of MDD in Fig. 1 after the most plausible social interaction effects have been determined, we apply a two-step approach to the resulting linear-in-means model. In Step 1, the outcome variable is modelled as a binary variable in a probit model, coded 1 for participants diagnosed with MDD based on one core and four or more secondary symptoms and 0 otherwise. In Step 2, the outcome variable is modelled as a continuous variable based on the total number of symptoms and estimated only for participants diagnosed with MDD. Using this two-step model, we can identify the extent to which individual and neighbourhood characteristics, as well as social interaction effects, contribute to the diagnosis of MDD on the one hand and to its severity on the other. To facilitate the interpretation and comparability of the estimation results of the probit model with those of linear regression models, we report only its Average Marginal Effects (AMEs). The estimates were performed in R [35].

## Results

Table 1 reports descriptive statistics of the individual and neighbourhood characteristics in the baseline assessment. The average age is 44.67 years (SD = 9.67), with most participants being female, Dutch-born, and having middle to high education. Most participants cohabit with their partner and children, report a good social circle and are non-smokers. On average, participants report 31.23 paid working hours per week. The average home value at the neighbourhood level is €196,640. The sample shows a slightly higher proportion of Western (non-Dutch) than non-Western populations, with most participants living in rural areas. The average distance to a GP is 5.31 km (SD = 7.24), indicating variability in healthcare access. The reported percentages of MDD of different categories in the case of discrete characteristics also differ significantly from each other.

**Table 1:** Descriptive statistics in the baseline assessment: Mean and standard deviation (SD) for continuous characteristics, and mean and percentage of MDD for discrete ones

	Baseline assessment (*n* = 19,700)	MDD (%)
*Individual characteristics*		
Age (years), mean (SD)	44.67 (9.67)	
Sex: Female	60.26	3.80
Male	39.74	2.08
Educational attainment: Low	25.26	4.92
Middle	40.40	2.89
High	33.58	1.74
Migration background: Born in the NL	97.53	2.97
Born outside the NL	2.47	8.62
Paid working hours per week, mean (SD)	31.23 (12.67)	
Social circle: Good	85.78	2.18
Fair	12.81	7.45
Poor	1.28	18.28
Partner status: No partner/no cohabitation	17.91	5.52
Partner/cohabiting	81.98	2.58
Children: No children/no cohabitation	43.46	3.50
Children/cohabiting	56.25	2.78
Smoking: Yes	19.15	4.99
No	80.65	2.64
*Neighbourhood characteristics*		
% Western population	5.62 (2.72)	
% Non-Western population	3.70 (4.25)	
Urbanity level: Rural	66.13	2.76
Moderate	17.13	4.30
High	16.74	3.31
Average home value (× 1,000 EUR)	196.64 (53.35)	
Average number of GP within 3 km	5.31 (7.24)	

The reported percentages of MDD of different categories per discrete covariate differ significantly from each other, either based on Welch’s *t*-test for two categories or on the Kruskal–Wallis test for more than two categories

The results of the Bayesian comparison test, applied to the linear-in-means model without peer effects, correlated effects, or both, are reported in Table 2. With a posterior model probability of 0.9988 in the first wave and 0.9991 in the second wave, a linear-in-means model with contextual and correlated effects is 900 to 1250 times more likely than its counterpart with contextual and peer effects. Apart from a few studies on depression [20], there is also generally little evidence of peer effects on MDD in adults. One possible explanation is that MDD represents an extreme manifestation of depression, typically characterised by a high degree of isolation. Consequently, not only is the presence of MDD less frequently observed, but individuals diagnosed with MDD also tend to have less interpersonal contact, which in turn influences the likelihood of peer effects.

**Table 2:** Results Bayesian comparison test for both waves

	First wave		Second wave	
	Log-marginal likelihood	Model probabilities	Log-marginal likelihood	Model probabilities
Linear-in means model without peer andcorrelated effects	− 40,254.67	0.0001	− 42,330.02	0.0001
Linear-in means model without correlated effects	− 40,007.44	0.0011	− 42,111.89	0.0008
Linear-in means modelwithout peer effects	− 39,993.91	0.9988	− 42,097.94	0.9991

Table 3 presents the estimation results of the two-step approach applied to the linear-in-means model (Model 1), comprising Step 1 and Step 2. The covariates in this model include individual and neighbourhood characteristics, referred to as “Own characteristics”, as well as contextual and correlated effects, referred to as “Social interaction effects”. Step 1 reports the AMEs estimated using a probit model based on a pooled sample of two cross-sections of 19,700 participants in both waves. Step 2 reports parameter estimates that can be interpreted in a way similar to AMEs. These estimates were obtained from a linear regression model based on a pooled sample of 614 participants from the first wave and 689 participants from the second wave. For comparison, and to assess the implications of accounting for the right-skewed distribution of MDD, particularly differences between diagnosis and severity, we also report the results of a naïve specification (Model 2) that does not incorporate this distinction between both steps. As in Step 2 of Model 1, Model 2 provides parameter estimates from a linear regression model, and as in Step 1 of Model 1, these estimates are based on pooled data from two repeated cross-sections of 19,700 participants.

**Table 3:** Estimated effects^§^ and standard errors (in parentheses) of the covariates

Covariates	Model 1: Two-step regression model				Model 2: Naïve model	
	Step 1 Probability MDD diagnosis (*n* = 39,400)		Step 2 Severity MDD diagnosis (*n* = 1303)		No distinction between Step 1 and 2 (*n* = 39,400)	
	Own characteristics	Social interaction effects	Own characteristics	Social interaction effects	Own characteristics	Social interaction effects
Intercept	− 1.91^+^ (0.98)		7.15** (0.84)		1.14** (0.26)	
*Individual characteristics*						
Sex (ref, Female): Male	− 0.002 (0.002)	− 0.01 (0.02)	− 0.04 (0.07)	− 0.84* (0.38)	− 0.11** (0.02)	− 0.01 (0.11)
Age	− 0.002 (0.001)	− 0.02 (0.04)	0.81* (0.35)	− 0.57 (1.15)	− 0.001 (0.08)	− 0.11 (0.36)
Educ. Level (ref, Low): Middle	− 0.004^+^ (0.002)	− 0.02^+^ (0.01)	− 0.14^+^ (0.07)	− 0.04 (0.36)	− 0.12** (0.02)	− 0.23* (0.09)
Educ. Level (ref, Low): High	− 0.01** (0.002)	− 0.01 (0.01)	− 0.06 (0.09)	− 0.32 (0.31)	− 0.18** (0.02)	− 0.28** (0.09))
Migration background (ref, the NL): outside the NL	0.02** (0.01)	− 0.03 (0.03)	− 0.23 (0.15)	0.09 (1.11)	0.22** (0.05)	− 0.16 (0.29)
Paid work hours per week	− 0.06** (0.01)	− 0.08^+^ (0.04)	− 0.09 (0.24)	2.36^+^ (1.32)	− 0.69** (0.07)	− 0.39 (0.34)
Partner status (ref, cohabiting): No	0.02** (0.003)	< − 0.001 (0.01)	0.10 (0.07)	− 0.78^+^ (0.41)	0.27** (0.02)	0.06 (0.12)
Children (ref, cohabiting): No	< 0.001 (0.002)	0.02* (0.01)	0.09 (0.07)	− 0.19 (0.26)	0.01 (0.02)	0.12 (0.08)
Social circle (ref, Good): Fair	0.05** (0.01)	0.01 (0.01)	0.16* (0.07)	0.42 (0.42)	0.73** (0.02)	0.23^+^ (0.13)
Social circle (ref, Good): Poor	0.11** (0.02)	0.08* (0.03)	0.52** (0.12)	0.21 (1.12)	1.65** (0.07)	0.83* (0.37)
Smoking (ref, No): Yes	0.01** (0.003)	0.01 (0.01)	− 0.04 (0.07)	− 0.47 (0.36)	0.21** (0.02)	0.21* (0.10)
Past depression (ref, No): Yes	0.04** (0.004)	0.06** (0.01)	− 0.02 (0.07)	0.79* (0.38)	0.59** (0.02)	0.91** (0.12)
*Neigbhourhood characteristics*						
% Western population	0.09 (0.06)		1.32 (1.68)		0.43 (0.51)	
% non-Western population	0.01 (0.03)		− 0.68 (0.95)		0.09 (0.29)	
Urbanity level (ref, High): Rural	− 0.001 (0.01)		0.07 (0.16)		0.01 (0.05)	
Urbanity level (ref, High): Moderate	< −0.001 (0.01)		− 0.11 (0.14)		0.04 (0.05)	
Average home value	− 0.08* (0.03)		− 1.64^+^ (0.95)		− 0.87** (0.24)	
Average number of GP within 3 km	− 0.54 (1.61)		4.15 (8.38)		− 3.39 (2.61)	
% of single person households	− 0.01 (0.03)		0.20 (0.47)		− 0.01 (0.14)	
*Random effects*						
Random effect (wave)	0.02** (0.01)		< 0.001**(0.004)		0.01** (0.001)	
Correlated effects		0.17** (0.04)		0.04** (0.02)		0.03** (0.02)

+ *p* < 0.1, *p < 0.05, **p < 0.01. § **Model 1**, **Step 1** Average Marginal Effects (AME) of probit random effects model, **Step 2** Parameter estimates of random effects model based on participants diagnosed with MDD. Model 2: **Naïve** random-effects **model** making no distinction between Step 1 and 2

The first important finding is that the results differ not only between the naïve and two-step models, but also between Step 1 and Step 2. The former is driven by the observation that most characteristics have larger parameter estimates in the naïve model than in either step of the two-step model, indicating that the naïve model tends to overestimate their magnitude. The latter is driven by the observation that many individual characteristics significantly associated with the diagnosis of MDD (6 out of 12) are not significantly associated with its severity. Together, these results highlight the importance of distinguishing between the diagnosis and severity of MDD.

The second finding is that the number of significant social interaction effects is substantially higher than the number of significant neighbourhood characteristics. Of the seven neighbourhood characteristics considered, only the average home value appears to have a significant impact on the probability of being diagnosed with MDD (AME = − 0.08, *p* < 0.05) and a weakly significant impact on its severity (− 1.64, *p* < 0.1). In contrast, five contextual effects significantly affect the probability of being diagnosed with MDD (Step 1, column “Social interactions effects”), and three affect its severity (Step 2, column “Social interactions effects”). The results of Step 1 further show that participants surrounded by people with secondary education (Education level: Middle) and/or more paid work hours per week are less likely to be diagnosed with an MDD episode (AME = − 0.02, *p* < 0.1; AME = − 0.08, *p* < 0.1, respectively). Conversely, participants surrounded by childless neighbours (AME = 0.02, *p* < 0.05) with poorer social ties (AME = 0.08, *p* < 0.05) and a history of depression are at greater risk of being diagnosed with MDD (AME = 0.06, *p* < 0.01). The results of Step 2, in turn, show that participants diagnosed with MDD surrounded by neighbours with a history of this depression (0.79, *p* < 0.05) are particularly vulnerable, unless they are male (− 0.84, *p* < 0.05) or do not cohabit with a partner (− 0.78, *p* < 0.1). Moreover, both steps of Model 1 show significant correlated effects between the error terms, which align with the posterior model probabilities from the Bayesian comparison test in Table 2. This suggests that shared unobserved characteristics within the neighbourhood, such as stigma or environmental stressors, influence both the diagnosis and the severity of MDD.

The third and last finding is that the individual characteristics remain the strongest determinants of MDD. Nine of them significantly contribute to the diagnosis of MDD, and four of these also affect its severity (Table 3, column “Own characteristics” of both Step 1 and Step 2). For example, people with a fair or poor social circle, rather than a good one, are at greater risk of being diagnosed with MDD (AME = 0.05, *p* < 0.01 and AME = 0.11, *p* < 0.01, respectively), as well as more severely affected (0.16, *p* < 0.05 and 0.52, *p* < 0.01, respectively).

## Discussion

This study provides insights into the contribution of social interaction effects to MDD. Using a Bayesian comparison approach, contextual and correlated effects are found to be empirically relevant, whereas peer effects are not. This may reflect the fact that MDD is an extreme manifestation of depression, typically characterised by a high degree of social isolation, which limits opportunities for peer influence.

Contextual and correlated effects not only appear to be significant determinants of MDD but also, in number, outweigh neighbourhood characteristics. While contextual effects are often interpreted as neighbourhood effects, in this study, we treat them as conceptually distinct: contextual effects reflect the average characteristics of other participants in the same neighbourhood, while neighbourhood effects are defined as characteristics based on aggregated data collected by an external statistical agency. When there is little overlap between both, adding contextual effects may capture more relevant variation than including only neighbourhood characteristics. For instance, we find the average paid hours per week among nearby individuals to have a negative spillover effect on the probability of being diagnosed with MDD and a positive spillover effect on its severity. One possible explanation is that an increase in paid working hours is associated with higher income, which reduces the likelihood of a diagnosis of MDD, but at the same time increases exposure to work-related stress. Such stress may spread through social networks via social contagion, thereby intensifying the severity of MDD [21, 36].

In line with previous research [15], we also find that the perception of a poor social circle by nearby people contributes significantly to the risk of MDD. A possible explanation is that this perception leads to reduced social cohesion within the neighbourhood, thereby increasing the risk of MDD. In contrast, a higher educational level (Middle and/or High relative to Low) of nearby people is associated with a lower risk of MDD, which is consistent with social gradient theory and previous studies showing that social cohesion and place attachment are more prevalent among people with a higher educational level [37].

The finding that correlated effects also contribute significantly to MDD may be partly related to the broader macroeconomic context at the time the data were collected. The first wave, during the period 2007–2013, coincided with the global financial crisis of 2008–2009 and the subsequent Great Recession, while the second wave, during the period 2014–2017, occurred at the end of the European debt crisis of 2010–2015, when unemployment peaked. These periods were characterised by increased financial stress and greater local differences in mental health outcomes [38], which may also partly explain the significance of the correlated effects.

## Limitations

Our study has some potential limitations that should be noted. First, participants’ responses in the sample may be influenced by self-reported bias. For example, people diagnosed with MDD may systematically perceive their social relationships more negatively than others. Second, instead of pooling the data over two waves, the model may also be estimated separately for each wave. The results of this robustness check are reported in Tables A3 and A4. Although the parameter estimates and the number of significant individual and neighbourhood characteristics, as well as social interaction effects, show some variation from the pooled analysis, the three main findings discussed earlier remain. Third, when pooling the data, it is also possible to control for individual random effects. However, this study opted for a cross-sectional approach, firstly to compare the pooled results in Table 2 with those of the individual waves in Tables A3 and A4, and secondly because the Bayesian comparison approach does not yet account for these random effects. Future research focusing on individual changes using a longitudinal design could further explore their influence. Lastly, it can be argued that the quality of the social circle, a key explanatory variable, might be endogenous. However, when the model is re-estimated without this variable, as shown in Table A5, the results again do not change substantially. This indicates that the possible endogeneity bias may be limited.

## Conclusion

This study shows that social interaction effects, particularly contextual and correlated effects, play an important role in shaping both the likelihood and severity of MDD among adults. To date, most social interaction studies have focused on peer effects because of the multiplier effects they have on others. However, contextual effects, the individual characteristics of nearby people, also spill over from one individual to another, albeit only locally within one’s own neighbourhood. Our findings show that in the case of MDD, a less frequently observed but extreme expression of depression, the number of significant contextual effects exceeds the number of significant neighbourhood characteristics. These include factors such as education level, paid working hours, social circle quality, household composition, and the depression history of nearby neighbours. Furthermore, this study shows that applying a two-step regression model provides better insights into the likelihood and severity of MDD. From a policy perspective, this distinction is of great importance in preventing overestimation of certain determining factors and to work more targeted in both steps. Overall, this study highlights the importance of addressing the social dimension of the neighbourhood in policies aimed at alleviating MDD.

## Supplementary Information

Below is the link to the electronic supplementary material.

ESM 1 (PDF 278 KB)

## Data Availability

Data may be obtained from a third party and is not publicly available. More information about how to apply for the data used in this study and the conditions of use can be found on the Lifelines website.
