# Supplementary material for: The Impact of Neighbours and Neighbourhoods on Major Depressive Disorders in Adults
Source: J Urban Health. 2026 Feb 10;103(2):370–81. doi: 10.1007/s11524-025-01055-x (PMC13235667; doi:10.1007/s11524-025-01055-x)
Supplement: Supplementary file 1 — (PDF 278 KB) [file 11524_2025_1055_MOESM1_ESM.pdf]

Appendix

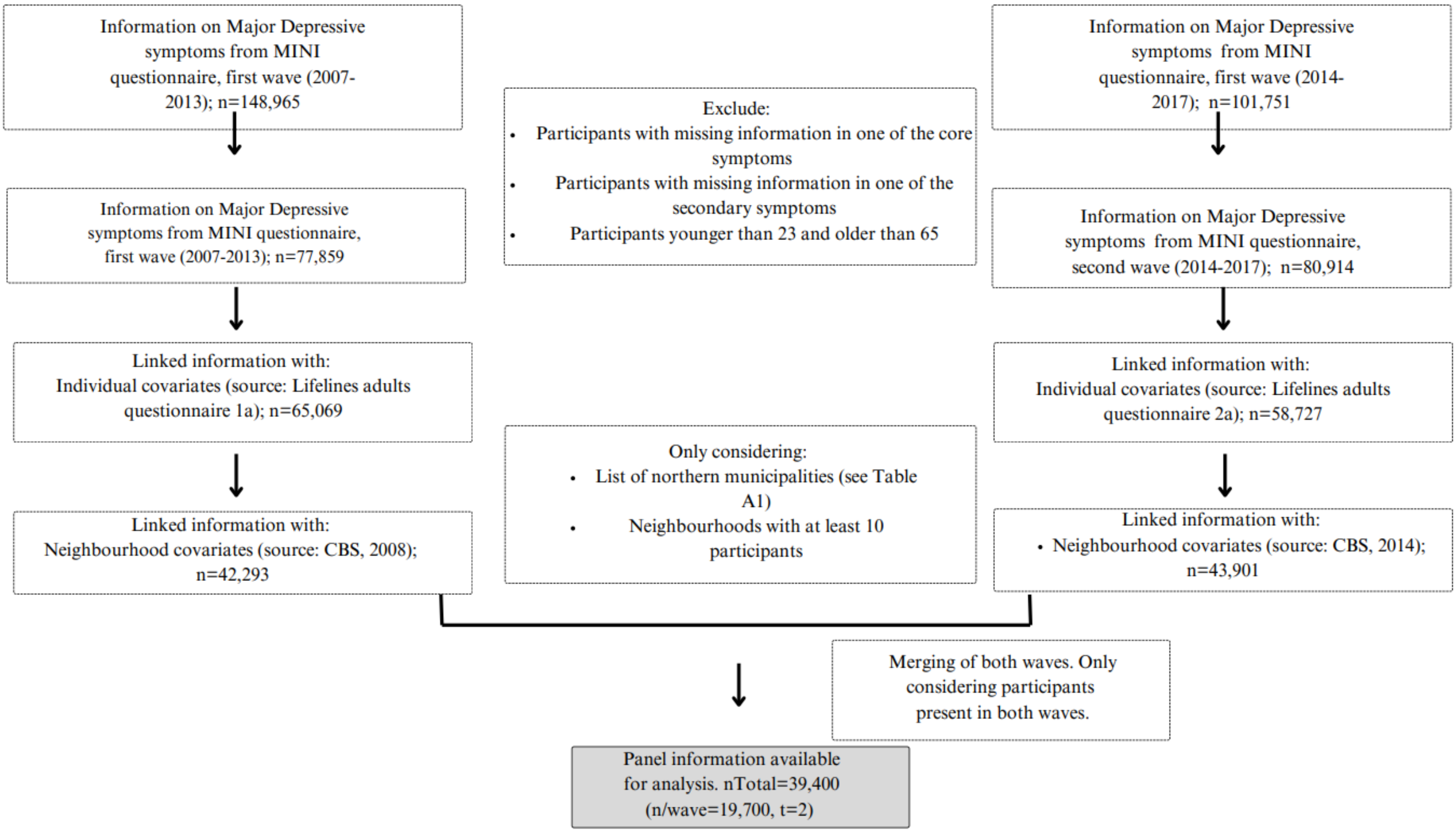

Figure A1. Flowchart of sample selection and inclusion criteria for both waves

Table A1. **S**elected municipalities (alphabetically ordered) in Northern Netherlands to analyse the association between neighbourhood characteristics and MDD

- Aa en Hunze, Achtkarspelen, Ameland, Appingedam, Assen, Bellingwedde
- Boarnsterhim, Borger-Odoorn
- Coevorden
- Dalfsen, Dantumadiel, De Marne, De Wolden, Delfzijl
- Eemsmon, Emmen
- Ferwerderadiel, Franekeradeel
- Gaasterlân-Sleat, Groningen, Grootegast
- Hardenberg, Harderwijk, Harlingen, Heerenveen, Hellendoorn, Hengelo, het Bildt, Hoogeveen Hoogezand-Sappemer
- Kampen, Kollumerland en Nieuwkruisland
- Leek, Leeuwarden, Leeuwarderadeel, Lemsterland, Littenseradiel, Loppersum
- Marum, Menameradiel, Menterwolde, Meppel, Midden-Drenthe
- Noordenveld
- Oldambt, Opsterland
- Pekela
- Raalte
- Skarsterlân, Slochteren, Smallingerland, Sûdwest Fryslân, Stadskanaal, Staphorst
- Ten Boer, Terschelling, Twenterland, Tynaarlo, Tytsjerksteradiel
- Veendam
- Vlagtwedde, Westerveld, Westervoort, Weststellingwerf, Zuidhorn, Zwartewaterland, Zwolle

Table A2. Questions to measure MDD in the MINI-questionnaire from the Lifelines Cohort Study

**Core symptoms**	**Question***
Consistently depressed	Have you been consistently depressed or down, most of the day, nearly every, day?
Low interest	In the past two weeks, have you been much less interested in most things or much less able to enjoy the things you used to enjoy most of the time?
**Secondary symptoms**	
Change in appetite and weight	Was your appetite decreased or increased nearly every day? Has your weight increased or decreased, although this was not the intention?
Insomnia	Did you have trouble sleeping nearly every night (difficulty falling asleep, waking up in the middle of the night, early morning wakening or sleeping excessively)?
Psychomotor retardation	Did you talk or move more slowly than normal, or were you fidgety, restless or having trouble sitting still almost every day?
Fatigue or energy loss	Did you feel tired or without energy almost every day?
Feeling of worthlessness or excessive guilt	Did you feel worthless or guilty almost every day?
Low concentration or decision-making	Did you have difficulty concentrating or making decisions every day?
Recurrent thoughts of death, suicidal ideation, plan or attempt	Did you repeatedly consider hurting yourself, feel suicidal, or wish that you were dead?

*All questions are framed in the past two weeks, participants can answer with *yes* (1) or *no* (0). A major depressive episode is established when at least one core symptom and at least five symptoms in total are present.

Table A3. Estimated effects[§] and standard errors (in parentheses) of the covariates for the first wave

	**Model 1: Two-step regression model**				**Model 2: Naïve model**	
	**Step 1 Probability MDD diagnosis** (n=19,700)		**Step 2 Severity MDD diagnosis** (n=614)		**No distinction between Step 1 and 2** (n=19,700)	
Covariates	Own characteristics	Social interaction effects	Own characteristics	Social interaction effects	Own characteristics	Social interaction effects
Intercept	-1.68 (1.37)		8.37** (1.37)		1.07** (0.36)	
Individual characteristics						
Sex (ref, Female): Male	-0.004 (0.003)	0.004 (0.01)	-0.06 (0.11)	-0.66 (0.58)	-0.10** (0.02)	0.09 (0.15)
Age	-0.03* (0.01)	-0.005 (0.05)	0.42 (0.54)	-2.49 (2.01)	-0.18+ (0.10)	0.13 (0.54)
Educ. Level (ref, Low): Middle	-0.01** (0.003)	-0.04** (0.01)	-0.22* (0.10)	-0.07 (0.55)	-0.27** (0.02)	-0.37* (0.15)
Educ. Level (ref, Low): High	-0.02** (0.003)	-0.023+ (0.012)	-0.02 (0.13)	-0.06 (0.44)	-0.37** (0.03)	-0.44** (0.13)
Migration background (ref, the NL): outside the NL	0.05** (0.01)	0.04 (0.04)	-0.07 (0.18)	-1.67 (1.72)	0.45** (0.06)	0.22 (0.45)
Paid work hours per week	-0.04** (0.01)	-0.06 (0.05)	-0.10 (0.36)	1.25 (1.94)	-0.45** (0.09)	-0.58 (0.51)
Partner status (ref, cohabiting): No	0.01** (0.003)	0.01 (0.02)	0.04 (0.11)	-0.55 (0.59)	0.18** (0.03)	0.08 (0.17)
Children (ref, cohabiting): No	-0.002 (0.002)	0.04** (0.01)	0.10 (0.09)	0.29 (0.38)	-0.03 (0.02)	0.41** (0.12)
Social circle (ref, Good): Fair	0.02** (0.004)	0.01 (0.02)	0.07 (0.10)	-0.37 (0.55)	0.48** (0.03)	0.15 (0.18)
Social circle (ref, Good): Poor	0.08** (0.01)	0.08* (0.05)	0.29+ (0.17)	-0.19 (1.80)	1.31** (0.08)	1.58** (0.54)
Smoking (ref, No): Yes	0.01* (0.003)	0.01 (0.01)	-0.19+ (0.10)	-1.14* (0.51)	0.12** (0.02)	0.10 (0.15)
Past depression (ref, No): Yes	0.08** (0.01)	0.07** (0.02)	0.26** (0.09)	1.09+ (0.65)	1.30** (0.03)	1.12** (0.19)
Neigbhourhood characteristics						
% Western population	-0.03 (0.07)		1.37 (2.69)		-0.45 (0.82)	
% non-Western population	0.01 (0.04)		0.70 (1.55)		0.06 (0.48)	
Urbanity level (ref, High): Rural	-0.003 (0.007)		-0.19 (0.26)		-0.02 (0.08)	
Urbanity level (ref, High): Moderate	0.002 (0.006)		-0.23 (0.23)		0.08 (0.08)	
Average home value	-0.09* (0.04)		-1.28 (1.47)		-0.59+ (0.35)	
Average number of GP within 3 km	-0.005 (0.004)		-0.12 (0.14)		-0.04 (0.04)	
% of single person households	-0.04* (0.02)		-0.54 (0.74)		-0.22 (0.22)	
Random effects						
Correlated effects		0.27** (0.51)		0.08 (0.28)		0.07** (0.27)

+p<0.1, *p<0.05, **p<0.01. § **Model 1**, **Step 1** Average Marginal Effects (AME) of probit random effects model, **Step 2** Parameter estimates of random effects model based on participants diagnosed with MDD. Model 2: **Naïve** random-effects **model** making no distinction between Step 1 and 2.

Table A4. Estimated effects§ and standard errors (in parentheses) of the covariates for the second wave

	**Model 1: Two-step regression model**				**Model 2: Naïve model**	
	**Step 1**<br>**Probability MDD diagnosis**<br>(n=19,700)		**Step 2**<br>**Severity MDD diagnosis**<br>(n=689)		**No distinction between Step 1 and 2**<br>(n=19,700)	
Covariates	Own characteristics	Social interaction effects	Own characteristics	Social interaction effects	Own characteristics	Social interaction effects
Intercept	-2.96* (1.23)		5.79** (1.20)		0.91** (0.35)	
Individual characteristics						
Sex (ref, Female): Male	0.001 (0.002)	-0.01 (0.02)	-0.02 (0.09)	-1.06* (0.50)	-0.10** (0.02)	-0.19 (0.15)
Age	0.003 (0.01)	-0.02 (0.04)	1.13* (0.47)	0.78 (1.76)	-0.13 (0.12)	0.20 (0.52)
Educ. Level (ref, Low): Middle	0.001* (0.003)	-0.02 (0.01)	-0.11 (0.11)	-0.29 (0.50)	0.02 (0.03)	-0.12 (0.14)
Educ. Level (ref, Low): High	0.01 (0.003)	-0.01 (0.01)	-0.14 (0.11)	-0.55 (0.44)	-0.02 (0.03)	-0.12 (0.11)
Migration background (ref, the NL): outside the NL	0.001** (0.01)	-0.03+ (0.03)	-0.58* (0.25)	1.33 (1.50)	0.01 (0.07)	-0.39 (0.40)
Paid work hours per week	-0.04** (0.01)	-0.08+ (0.04)	0.09 (0.34)	4.32* (1.95)	-0.66** (0.10)	0.61 (0.52)
Partner status (ref, cohabiting): No	0.02** (0.003)	<-0.001 (0.01)	0.13 (0.09)	-1.17* (0.57)	0.32** (0.03)	-0.08 (0.17)
Children (ref, cohabiting): No	0.001 (0.002)	0.02 (0.01)	0.09 (0.08)	-0.54 (0.38)	0.03 (0.02)	-0.06 (0.11)
Social circle (ref, Good): Fair	0.07** (0.01)	0.01 (0.01)	0.22* (0.09)	1.56* (0.66)	0.94** (0.04)	0.22 (0.19)
Social circle (ref, Good): Poor	0.15** (0.02)	0.08 (0.03)	0.85** (0.18)	0.92 (1.50)	1.84** (0.10)	0.55 (0.54)
Smoking (ref, No): Yes	0.02** (0.003)	0.01 (0.01)	0.09 (0.09)	0.19 (0.53)	0.25** (0.03)	0.28+ (0.15)
Past depression (ref, No): Yes	0.01* (0.004)	0.06** (0.01)	-0.35** (0.12)	0.52 (0.48)	0.04 (0.03)	1.04** (0.16)
Neighbourhood characteristics						
% Western population	0.13+ (0.07)		0.34 (2.19)		0.40 (0.69)	
% non-Western population	0.004 (0.04)		-0.67 (1.18)		0.17 (0.36)	
Urbanity level (ref, High): Rural	-0.001 (0.01)		0.22 (0.19)		0.01 (0.07)	
Urbanity level (ref, High): Moderate	-0.001 (0.01)		-0.003 (0.18)		0.005 (0.06)	
Average home value	-0.06 (0.04)		-1.54 (1.33)		-0.99** (0.36)	
Average number of GP within 3 km	-0.01+ (0.004)		0.17+ (0.18)		-0.02 (0.03)	
% of single person households	0.01 (0.02)		0.28 (0.60)		0.07 (0.18)	
Random effects						
Correlated effects		0.10** (0.32)		<0.001** (<0.001)		0.02** (0.15)

+p<0.1, *p<0.05, **p<0.01. § **Model 1**, **Step 1** Average Marginal Effects (AME) of probit random effects model, **Step 2** Parameter estimates of random effects model based on participants diagnosed with MDD. Model 2: **Naïve** random-effects **model** making no distinction between Step 1 and 2.

Table A5. Estimated effects§ and standard errors (in parentheses) of the covariates without the quality of the social circle

	Model 1: Two-step regression model				Model 2: Naïve model	
	**Step 1** **Probability MDD diagnosis** (n=39,400)		**Step 2** **Severity MDD diagnosis** (n=1,303)		**No distinction between Step 1 and 2** (n=39,400)	
Covariates	Own characteristics	Social interaction effects	Own characteristics	Social interaction effects	Own characteristics	Social interaction effects
Intercept	-1.98* (0.81)		7.30** (0.83)		1.31** (0.26)	
Individual characteristics						
Sex (ref, Female): Male	-0.003 (0.002)	-0.30 (0.39)	-0.04 (0.08)	-0.84* (0.38)	-0.13** (0.02)	-0.01 (0.11)
Age	-0.02* (0.01)	0.85 (1.12)	0.71* (0.35)	-0.77 (1.14)	-0.27** (0.08)	0.10 (0.37)
Educ. Level (ref, Low): Middle	-0.004+ (0.002)	-0.57 (0.36)	-0.15* (0.07)	-0.003 (0.36)	-0.12** (0.02)	-0.23* (0.10)
Educ. Level (ref, Low): High	-0.01* (0.002)	-0.43 (0.32)	-0.07 (0.09)	-0.32 (0.31)	-0.19** (0.02)	-0.32** (0.09)
Migration background (ref, the NL): outside the NL	0.02** (0.01)	-1.12 (1.11)	-0.25+ (0.15)	-0.07 (1.11)	0.21** (0.05)	-0.23 (0.30)
Paid work hours per week	-0.06** (0.01)	-2.21+ (1.28)	-0.12 (0.24)	2.50+ (1.32)	-0.78** (0.07)	-0.49 (0.35)
Partner status (ref, cohabiting): No	0.02** (0.003)	0.22 (0.42)	0.14* (0.08)	-0.70+ (0.41)	0.36** (0.02)	0.19 (0.12)
Children (ref, cohabiting): No	0.001 (0.002)	0.56* (0.28)	0.09 (0.07)	-0.15 (0.26)	0.01 (0.02)	0.12 (0.08)
Smoking (ref, No): Yes	0.02** (0.003)	0.39 (0.36)	-0.03 (0.07)	-0.42 (0.36)	0.24** (0.02)	0.22* (0.10)
Past depression (ref, No): Yes	0.05** (0.004)	2.44** (0.39)	-0.002 (0.07)	0.84* (0.37)	0.66** (0.02)	0.99** (0.12)
Neigbhourhood characteristics						
% Western population	0.08 (0.05)		1.18 (1.68)		0.34 (0.52)	
% non-Western population	0.02 (0.03)		-0.48 (0.95)		0.30 (0.29)	
Urbanity level (ref, High): Rural	<-0.001 (0.005)		0.08 (0.16)		0.02 (0.05)	
Urbanity level (ref, High): Moderate	<0.001 (0.004)		-0.10 (0.14)		0.06 (0.05)	
Average home value	-0.08** (0.03)		-1.63+ (0.96)		-0.85** (0.25)	
Average number of GP within 3 km	-0.005 (0.003)		0.04 (0.08)		-0.03 (0.03)	
% of single person households	-0.01 (0.01)		0.27 (0.47)		0.04 (0.14)	
Random effects						
Random effect (wave)	<0.001** (<0.001)		<0.001*(<0.001)		0.01** (<0.01)	
Correlated effects		0.18** (0.04)		0.04* (0.02)		0.04** (0.02)

+p<0.1, *p<0.05, **p<0.01. § **Model 1**, **Step 1** Average Marginal Effects (AME) of probit random effects model, **Step 2** Parameter estimates of random effects model based on participants diagnosed with MDD. Model 2: **Naïve** random-effects **model** making no distinction between Step 1 and 2.
